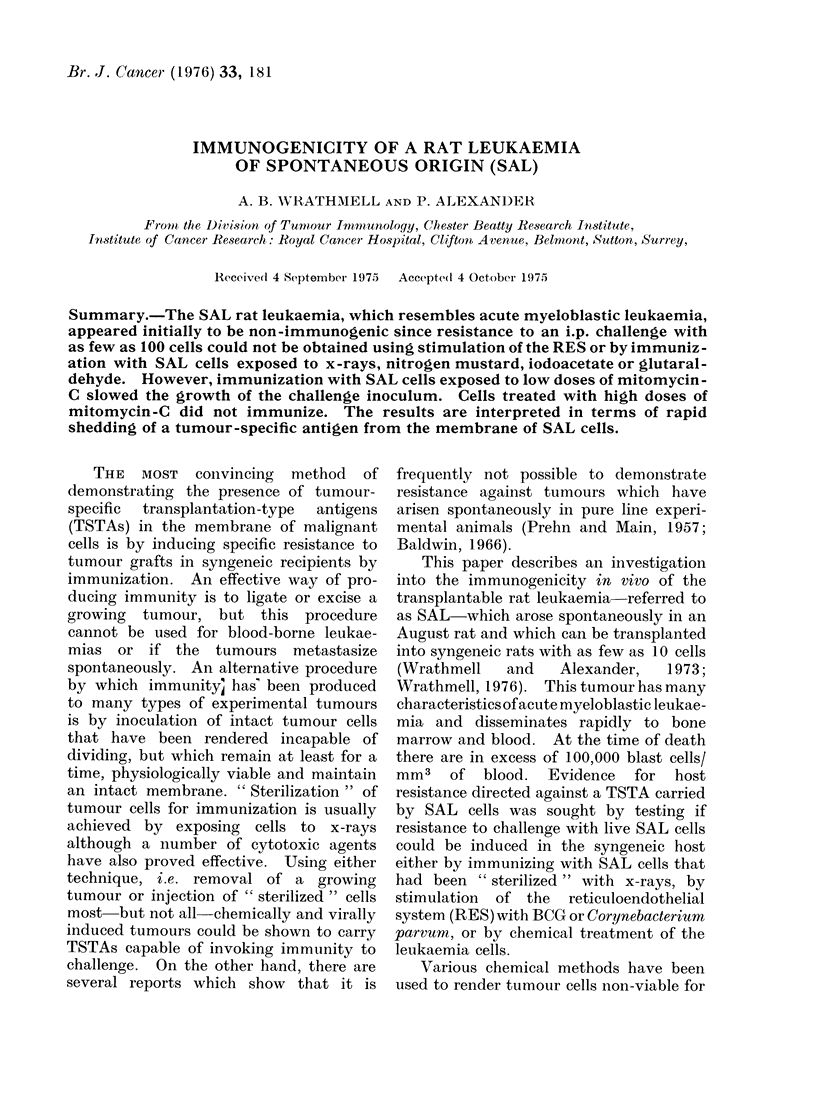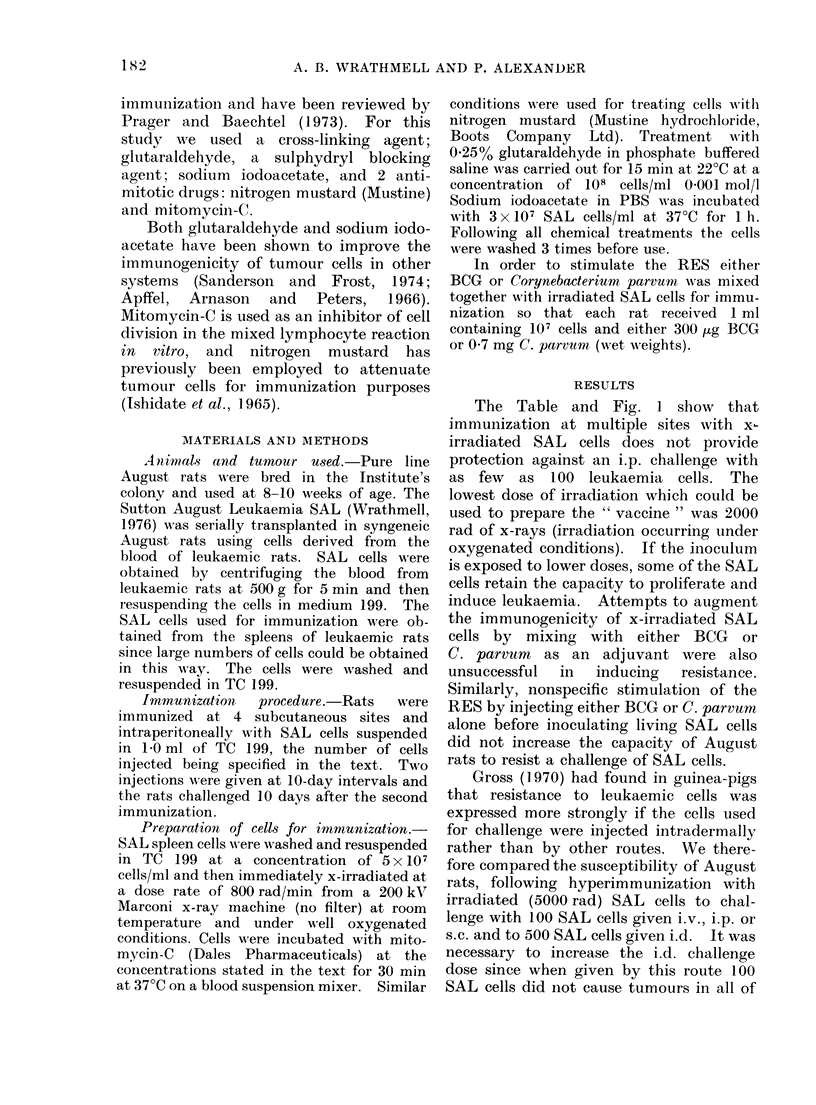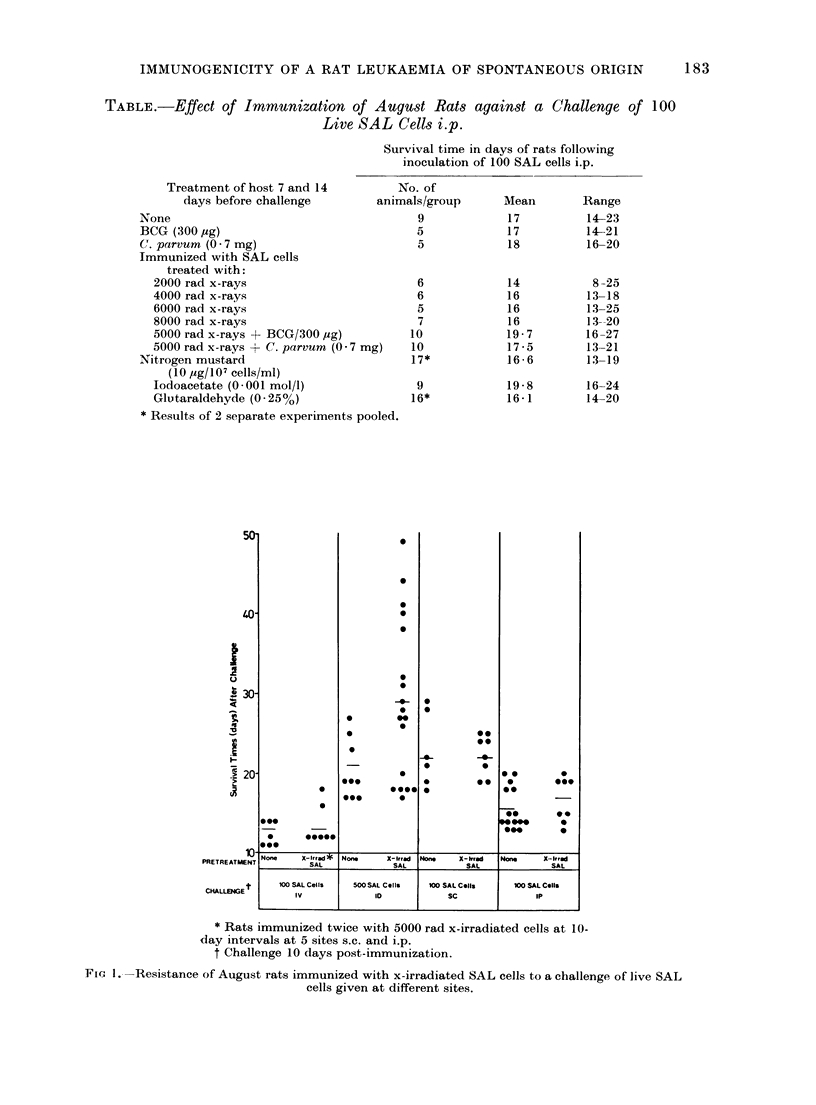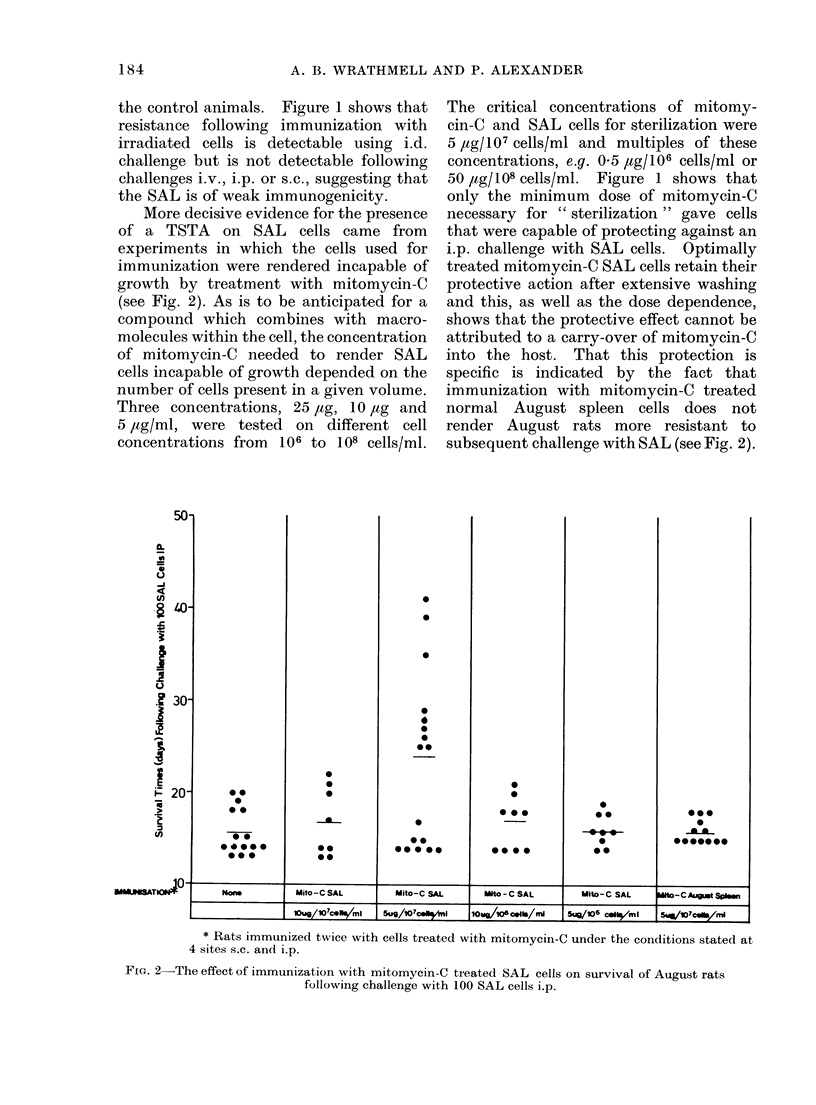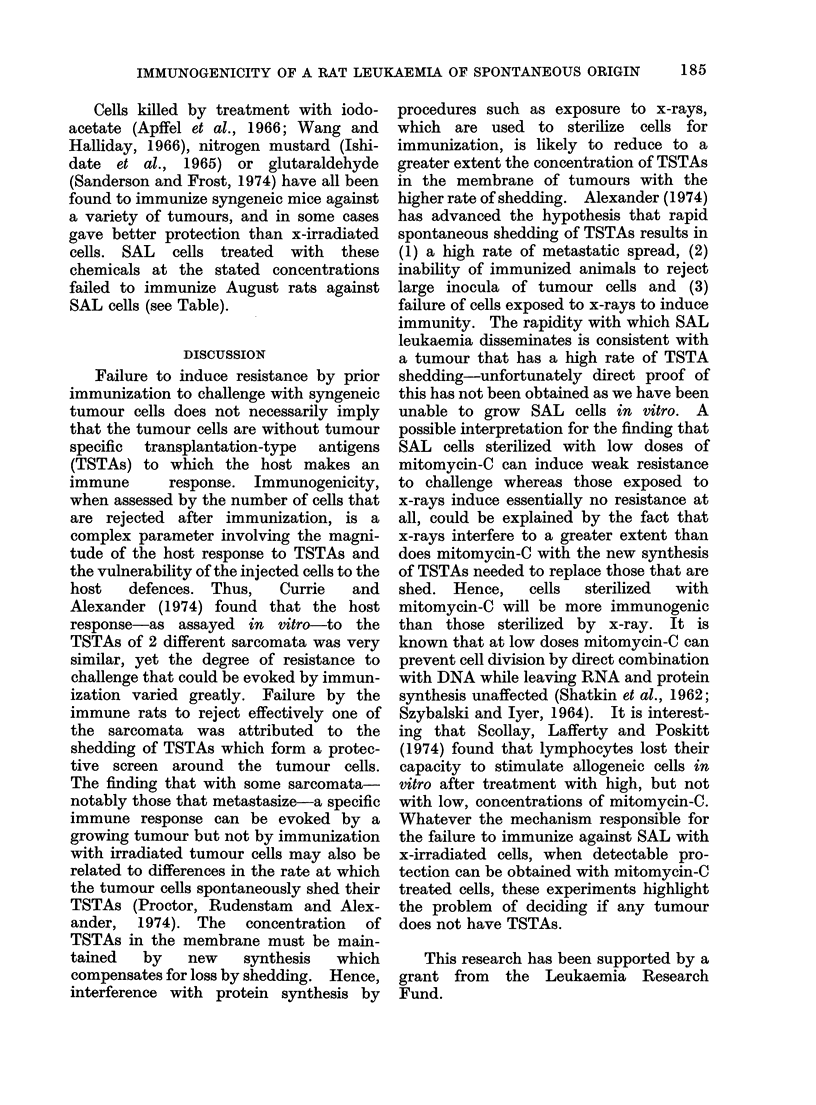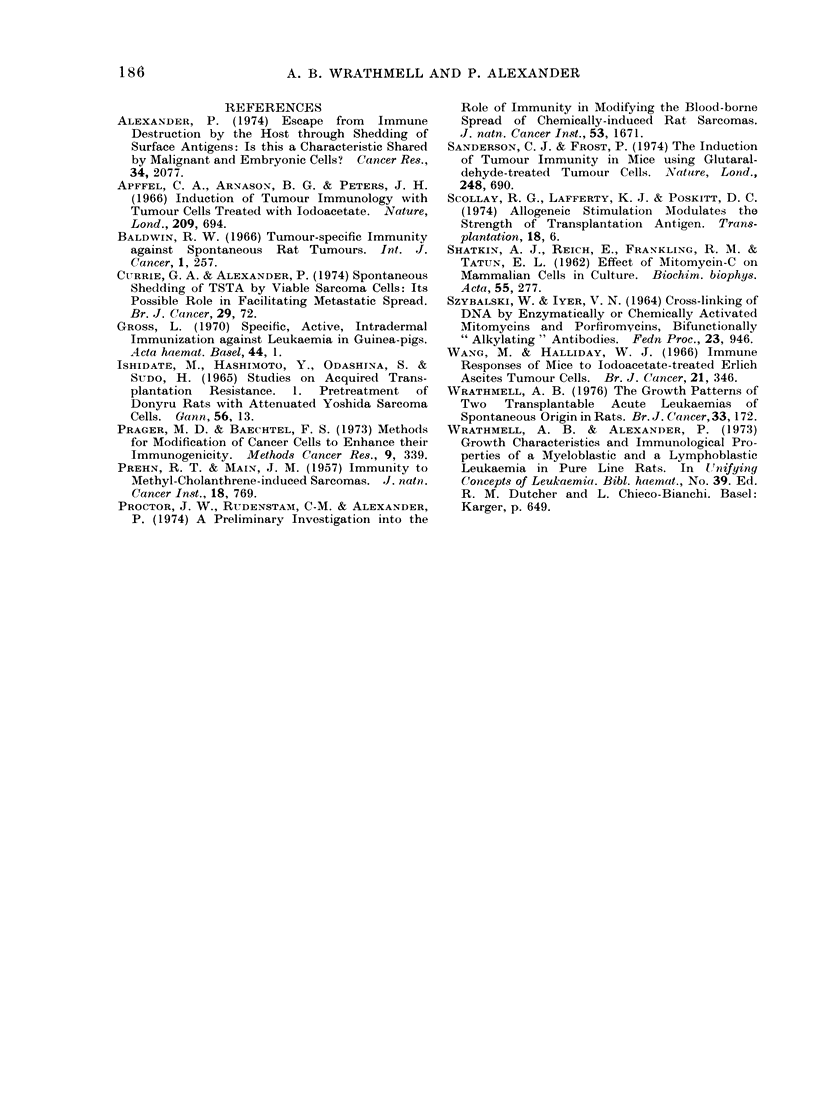# Immunogenicity of a rat leukaemia of spontaneous origin (SAL).

**DOI:** 10.1038/bjc.1976.23

**Published:** 1976-02

**Authors:** A. B. Wrathmell, P. Alexander

## Abstract

The SAL rat leukaemia, which resembles acute myeloblastic leukaemia, appeared initially to be non-immunogenic since resistance to an i.p. challenge with as few as 100 cells could not be obtained using stimulation of the RES or by immunization with SAL cells exposed to x-rays, nitrogen mustard, iodoacetate or glutaraldehyde. However, immunization with SAL cells exposed to low doses of mitomycin-C slowed the growth of the challenge inoculum. Cells treated with high doses of mitomycin-C did not immunize. The results are interpreted in terms of rapid shedding of a tumour-specific antigen from the membrane of SAL cells.


*Br. J. Cancer* (1976) **33**, 181

### IMMUNOGENICITY OF A RAT LEUKAEMIA OF SPONTANEOUS ORIGIN (SAL)

A. B. WRATHMELL AND P. ALEXANDER

*From the Division of Tumour Immunology, Chester Beatty Research Institute, Institute of Cancer Research: Royal Cancer Hospital, Clifton Avenue, Belmont, Sutton, Surrey,*

Received 4 September 1975 Accepted 4 October 1975

**Summary.—The SAL rat leukaemia, which resembles acute myeloblastic leukaemia, appeared initially to be non-immunogenic since resistance to an i.p. challenge with as few as 100 cells could not be obtained using stimulation of the RES or by immunization with SAL cells exposed to x-rays, nitrogen mustard, iodoacetate or glutaraldehyde. However, immunization with SAL cells exposed to low doses of mitomycin-C slowed the growth of the challenge inoculum. Cells treated with high doses of mitomycin-C did not immunize. The results are interpreted in terms of rapid shedding of a tumour-specific antigen from the membrane of SAL cells.**

THE MOST convincing method of demonstrating the presence of tumour-specific transplantation-type antigens (TSTAs) in the membrane of malignant cells is by inducing specific resistance to tumour grafts in syngeneic recipients by immunization. An effective way of producing immunity is to ligate or excise a growing tumour, but this procedure cannot be used for blood-borne leukaemias or if the tumours metastasize spontaneously. An alternative procedure by which immunity has been produced to many types of experimental tumours is by inoculation of intact tumour cells that have been rendered incapable of dividing, but which remain at least for a time, physiologically viable and maintain an intact membrane. " Sterilization " of tumour cells for immunization is usually achieved by exposing cells to x-rays although a number of cytotoxic agents have also proved effective. Using either technique, *i.e.* removal of a growing tumour or injection of " sterilized " cells most—but not all—chemically and virally induced tumours could be shown to carry TSTAs capable of invoking immunity to challenge. On the other hand, there are several reports which show that it is frequently not possible to demonstrate resistance against tumours which have arisen spontaneously in pure line experimental animals (Prehn and Main, 1957; Baldwin, 1966).

This paper describes an investigation into the immunogenicity *in vivo* of the transplantable rat leukaemia—referred to as SAL—which arose spontaneously in an August rat and which can be transplanted into syngeneic rats with as few as 10 cells (Wrathmell and Alexander, 1973; Wrathmell, 1976). This tumour has many characteristics of acute myeloblastic leukaemia and disseminates rapidly to bone marrow and blood. At the time of death there are in excess of 100,000 blast cells/$mm^3$ of blood. Evidence for host resistance directed against a TSTA carried by SAL cells was sought by testing if resistance to challenge with live SAL cells could be induced in the syngeneic host either by immunizing with SAL cells that had been " sterilized " with x-rays, by stimulation of the reticuloendothelial system (RES) with BCG or *Corynebacterium parvum*, or by chemical treatment of the leukaemia cells.

Various chemical methods have been used to render tumour cells non-viable for immunization and have been reviewed by Prager and Baechtel (1973). For this study we used a cross-linking agent; glutaraldehyde, a sulphydryl blocking agent; sodium iodoacetate, and 2 antimitotic drugs: nitrogen mustard (Mustine) and mitomycin-C.

Both glutaraldehyde and sodium iodoacetate have been shown to improve the immunogenicity of tumour cells in other systems (Sanderson and Frost, 1974; Apffel, Arnason and Peters, 1966). Mitomycin-C is used as an inhibitor of cell division in the mixed lymphocyte reaction *in vitro*, and nitrogen mustard has previously been employed to attenuate tumour cells for immunization purposes (Ishidate *et al.*, 1965).

### MATERIALS AND METHODS

*Animals and tumour used.*—Pure line August rats were bred in the Institute's colony and used at 8–10 weeks of age. The Sutton August Leukaemia SAL (Wrathmell, 1976) was serially transplanted in syngeneic August rats using cells derived from the blood of leukaemic rats. SAL cells were obtained by centrifuging the blood from leukaemic rats at 500 g for 5 min and then resuspending the cells in medium 199. The SAL cells used for immunization were obtained from the spleens of leukaemic rats since large numbers of cells could be obtained in this way. The cells were washed and resuspended in TC 199.

*Immunization procedure.*—Rats were immunized at 4 subcutaneous sites and intraperitoneally with SAL cells suspended in 1·0 ml of TC 199, the number of cells injected being specified in the text. Two injections were given at 10-day intervals and the rats challenged 10 days after the second immunization.

*Preparation of cells for immunization.*—SAL spleen cells were washed and resuspended in TC 199 at a concentration of $5 \times 10^7$ cells/ml and then immediately x-irradiated at a dose rate of 800 rad/min from a 200 kV Marconi x-ray machine (no filter) at room temperature and under well oxygenated conditions. Cells were incubated with mitomycin-C (Dales Pharmaceuticals) at the concentrations stated in the text for 30 min at 37°C on a blood suspension mixer. Similar conditions were used for treating cells with nitrogen mustard (Mustine hydrochloride, Boots Company Ltd). Treatment with 0·25% glutaraldehyde in phosphate buffered saline was carried out for 15 min at 22°C at a concentration of $10^8$ cells/ml 0·001 mol/l Sodium iodoacetate in PBS was incubated with $3 \times 10^7$ SAL cells/ml at 37°C for 1 h. Following all chemical treatments the cells were washed 3 times before use.

In order to stimulate the RES either BCG or *Corynebacterium parvum* was mixed together with irradiated SAL cells for immunization so that each rat received 1 ml containing $10^7$ cells and either 300 $\mu$g BCG or 0·7 mg *C. parvum* (wet weights).

### RESULTS

The Table and Fig. 1 show that immunization at multiple sites with x-irradiated SAL cells does not provide protection against an i.p. challenge with as few as 100 leukaemia cells. The lowest dose of irradiation which could be used to prepare the " vaccine " was 2000 rad of x-rays (irradiation occurring under oxygenated conditions). If the inoculum is exposed to lower doses, some of the SAL cells retain the capacity to proliferate and induce leukaemia. Attempts to augment the immunogenicity of x-irradiated SAL cells by mixing with either BCG or *C. parvum* as an adjuvant were also unsuccessful in inducing resistance. Similarly, nonspecific stimulation of the RES by injecting either BCG or *C. parvum* alone before inoculating living SAL cells did not increase the capacity of August rats to resist a challenge of SAL cells.

Gross (1970) had found in guinea-pigs that resistance to leukaemic cells was expressed more strongly if the cells used for challenge were injected intradermally rather than by other routes. We therefore compared the susceptibility of August rats, following hyperimmunization with irradiated (5000 rad) SAL cells to challenge with 100 SAL cells given i.v., i.p. or s.c. and to 500 SAL cells given i.d. It was necessary to increase the i.d. challenge dose since when given by this route 100 SAL cells did not cause tumours in all of

TABLE.—*Effect of Immunization of August Rats against a Challenge of* 100 *Live SAL Cells i.p.*

| Treatment of host 7 and 14 days before challenge | Survival time in days of rats following inoculation of 100 SAL cells i.p. No. of animals/group | Mean | Range |
|---|---|---|---|
| None | 9 | 17 | 14–23 |
| BCG (300 μg) | 5 | 17 | 14–21 |
| *C. parvum* (0·7 mg) | 5 | 18 | 16–20 |
| Immunized with SAL cells treated with: | | | |
| 2000 rad x-rays | 6 | 14 | 8–25 |
| 4000 rad x-rays | 6 | 16 | 13–18 |
| 6000 rad x-rays | 5 | 16 | 13–25 |
| 8000 rad x-rays | 7 | 16 | 13–20 |
| 5000 rad x-rays + BCG/300 μg) | 10 | 19·7 | 16–27 |
| 5000 rad x-rays + *C. parvum* (0·7 mg) | 10 | 17·5 | 13–21 |
| Nitrogen mustard (10 μg/$10^7$ cells/ml) | 17* | 16·6 | 13–19 |
| Iodoacetate (0·001 mol/l) | 9 | 19·8 | 16–24 |
| Glutaraldehyde (0·25%) | 16* | 16·1 | 14–20 |

* Results of 2 separate experiments pooled.

* Rats immunized twice with 5000 rad x-irradiated cells at 10-day intervals at 5 sites s.c. and i.p.
† Challenge 10 days post-immunization.

FIG 1.—Resistance of August rats immunized with x-irradiated SAL cells to a challenge of live SAL cells given at different sites.

the control animals. Figure 1 shows that resistance following immunization with irradiated cells is detectable using i.d. challenge but is not detectable following challenges i.v., i.p. or s.c., suggesting that the SAL is of weak immunogenicity.

More decisive evidence for the presence of a TSTA on SAL cells came from experiments in which the cells used for immunization were rendered incapable of growth by treatment with mitomycin-C (see Fig. 2). As is to be anticipated for a compound which combines with macromolecules within the cell, the concentration of mitomycin-C needed to render SAL cells incapable of growth depended on the number of cells present in a given volume. Three concentrations, 25 $\mu$g, 10 $\mu$g and 5 $\mu$g/ml, were tested on different cell concentrations from $10^6$ to $10^8$ cells/ml. The critical concentrations of mitomycin-C and SAL cells for sterilization were 5 $\mu$g/$10^7$ cells/ml and multiples of these concentrations, *e.g.* 0·5 $\mu$g/$10^6$ cells/ml or 50 $\mu$g/$10^8$ cells/ml. Figure 1 shows that only the minimum dose of mitomycin-C necessary for " sterilization " gave cells that were capable of protecting against an i.p. challenge with SAL cells. Optimally treated mitomycin-C SAL cells retain their protective action after extensive washing and this, as well as the dose dependence, shows that the protective effect cannot be attributed to a carry-over of mitomycin-C into the host. That this protection is specific is indicated by the fact that immunization with mitomycin-C treated normal August spleen cells does not render August rats more resistant to subsequent challenge with SAL (see Fig. 2).

* Rats immunized twice with cells treated with mitomycin-C under the conditions stated at 4 sites s.c. and i.p.

FIG. 2—The effect of immunization with mitomycin-C treated SAL cells on survival of August rats following challenge with 100 SAL cells i.p.

Cells killed by treatment with iodoacetate (Apffel *et al.*, 1966; Wang and Halliday, 1966), nitrogen mustard (Ishidate *et al.*, 1965) or glutaraldehyde (Sanderson and Frost, 1974) have all been found to immunize syngeneic mice against a variety of tumours, and in some cases gave better protection than x-irradiated cells. SAL cells treated with these chemicals at the stated concentrations failed to immunize August rats against SAL cells (see Table).

### DISCUSSION

Failure to induce resistance by prior immunization to challenge with syngeneic tumour cells does not necessarily imply that the tumour cells are without tumour specific transplantation-type antigens (TSTAs) to which the host makes an immune response. Immunogenicity, when assessed by the number of cells that are rejected after immunization, is a complex parameter involving the magnitude of the host response to TSTAs and the vulnerability of the injected cells to the host defences. Thus, Currie and Alexander (1974) found that the host response—as assayed *in vitro*—to the TSTAs of 2 different sarcomata was very similar, yet the degree of resistance to challenge that could be evoked by immunization varied greatly. Failure by the immune rats to reject effectively one of the sarcomata was attributed to the shedding of TSTAs which form a protective screen around the tumour cells. The finding that with some sarcomata—notably those that metastasize—a specific immune response can be evoked by a growing tumour but not by immunization with irradiated tumour cells may also be related to differences in the rate at which the tumour cells spontaneously shed their TSTAs (Proctor, Rudenstam and Alexander, 1974). The concentration of TSTAs in the membrane must be maintained by new synthesis which compensates for loss by shedding. Hence, interference with protein synthesis by procedures such as exposure to x-rays, which are used to sterilize cells for immunization, is likely to reduce to a greater extent the concentration of TSTAs in the membrane of tumours with the higher rate of shedding. Alexander (1974) has advanced the hypothesis that rapid spontaneous shedding of TSTAs results in (1) a high rate of metastatic spread, (2) inability of immunized animals to reject large inocula of tumour cells and (3) failure of cells exposed to x-rays to induce immunity. The rapidity with which SAL leukaemia disseminates is consistent with a tumour that has a high rate of TSTA shedding—unfortunately direct proof of this has not been obtained as we have been unable to grow SAL cells *in vitro*. A possible interpretation for the finding that SAL cells sterilized with low doses of mitomycin-C can induce weak resistance to challenge whereas those exposed to x-rays induce essentially no resistance at all, could be explained by the fact that x-rays interfere to a greater extent than does mitomycin-C with the new synthesis of TSTAs needed to replace those that are shed. Hence, cells sterilized with mitomycin-C will be more immunogenic than those sterilized by x-ray. It is known that at low doses mitomycin-C can prevent cell division by direct combination with DNA while leaving RNA and protein synthesis unaffected (Shatkin *et al.*, 1962; Szybalski and Iyer, 1964). It is interesting that Scollay, Lafferty and Poskitt (1974) found that lymphocytes lost their capacity to stimulate allogeneic cells *in vitro* after treatment with high, but not with low, concentrations of mitomycin-C. Whatever the mechanism responsible for the failure to immunize against SAL with x-irradiated cells, when detectable protection can be obtained with mitomycin-C treated cells, these experiments highlight the problem of deciding if any tumour does not have TSTAs.

This research has been supported by a grant from the Leukaemia Research Fund.

REFERENCES

ALEXANDER, P. (1974) Escape from Immune Destruction by the Host through Shedding of Surface Antigens: Is this a Characteristic Shared by Malignant and Embryonic Cells? *Cancer Res.*, **34,** 2077.

APFFEL, C. A., ARNASON, B. G. & PETERS, J. H. (1966) Induction of Tumour Immunology with Tumour Cells Treated with Iodoacetate. *Nature, Lond.*, **209**, 694.

BALDWIN, R. W. (1966) Tumour-specific Immunity against Spontaneous Rat Tumours. *Int. J. Cancer*, **1**, 257.

CURRIE, G. A. & ALEXANDER, P. (1974) Spontaneous Shedding of TSTA by Viable Sarcoma Cells: Its Possible Role in Facilitating Metastatic Spread. *Br. J. Cancer*, **29**, 72.

GROSS, L. (1970) Specific, Active, Intradermal Immunization against Leukaemia in Guinea-pigs. *Acta haemat. Basel*, **44**, 1.

ISHIDATE, M., HASHIMOTO, Y., ODASHINA, S. & SUDO, H. (1965) Studies on Acquired Transplantation Resistance. 1. Pretreatment of Donyru Rats with Attenuated Yoshida Sarcoma Cells. *Gann*, **56**, 13.

PRAGER, M. D. & BAECHTEL, F. S. (1973) Methods for Modification of Cancer Cells to Enhance their Immunogenicity. *Methods Cancer Res.*, **9**, 339.

PREHN, R. T. & MAIN, J. M. (1957) Immunity to Methyl-Cholanthrene-induced Sarcomas. *J. natn. Cancer Inst.*, **18**, 769.

PROCTOR, J. W., RUDENSTAM, C-M. & ALEXANDER, P. (1974) A Preliminary Investigation into the Role of Immunity in Modifying the Blood-borne Spread of Chemically-induced Rat Sarcomas. *J. natn. Cancer Inst.*, **53**, 1671.

SANDERSON, C. J. & FROST, P. (1974) The Induction of Tumour Immunity in Mice using Glutaraldehyde-treated Tumour Cells. *Nature, Lond.*, **248**, 690.

SCOLLAY, R. G., LAFFERTY, K. J. & POSKITT, D. C. (1974) Allogeneic Stimulation Modulates the Strength of Transplantation Antigen. *Transplantation*, **18**, 6.

SHATKIN, A. J., REICH, E., FRANKLING, R. M. & TATUN, E. L. (1962) Effect of Mitomycin-C on Mammalian Cells in Culture. *Biochim. biophys. Acta*, **55**, 277.

SZYBALSKI, W. & IYER, V. N. (1964) Cross-linking of DNA by Enzymatically or Chemically Activated Mitomycins and Porfiromycins, Bifunctionally " Alkylating " Antibodies. *Fedn Proc.*, **23**, 946.

WANG, M. & HALLIDAY, W. J. (1966) Immune Responses of Mice to Iodoacetate-treated Erlich Ascites Tumour Cells. *Br. J. Cancer*, **21**, 346.

WRATHMELL, A. B. (1976) The Growth Patterns of Two Transplantable Acute Leukaemias of Spontaneous Origin in Rats. *Br. J. Cancer*, **33**, 172.

WRATHMELL, A. B. & ALEXANDER, P. (1973) Growth Characteristics and Immunological Properties of a Myeloblastic and a Lymphoblastic Leukaemia in Pure Line Rats. In *Unifying Concepts of Leukaemia. Bibl. haemat.*, No. **39**. Ed. R. M. Dutcher and L. Chieco-Bianchi. Basel: Karger, p. 649.